# Macrolide resistance in *Mycoplasma pneumoniae* in adult patients

**DOI:** 10.3389/fcimb.2025.1496521

**Published:** 2025-03-04

**Authors:** Panpan Xie, Yue Zhang, Yanhong Qin, Yun Fang, Ning Yang, Yunbiao Bai, Shimeng Zhi, Wenkai Niu, Fusheng Wang, Xin Yuan

**Affiliations:** ^1^ Department of Respiratory and Critical Care Medicine, Senior Department of Infectious Diseases, the Fifth Medical Center of PLA General Hospital, Beijing, China; ^2^ The Fifth Clinical Medical College, Anhui Medical University, Hefei, Anhui, China; ^3^ Senior Department of Infectious Diseases, The Fifth Medical Center of PLA General Hospital, National Clinical Research Center for Infectious Diseases, Beijing, China

**Keywords:** *Mycoplasma pneumoniae*, macrolide resistance, resistant mechanism, point mutations, efflux pump

## Abstract

*Mycoplasma pneumoniae* is one of the most significant pathogens responsible for respiratory infections in humans. Macrolides are recommended as the first-line treatment for *M. pneumoniae* infection. The prevalence of macrolide-resistant *M. pneumoniae* has increased significantly in recent decades, particularly in China. The mechanisms of resistance in *M. pneumoniae* to macrolides have been extensively studied in pediatric patients. However, a paucity reports regarding the resistance characteristics and mechanisms exhibited in adults. The aim of this study was to elucidate the resistance of *M. pneumoniae* to macrolides and the underlying mechanisms in adult patients. Pharyngeal swab specimens were collected from adult patients presenting with subacute cough or community-acquired pneumonia at our hospital from January 2011 to June 2017 to identify and isolate *M. pneumoniae* strains. The antimicrobial susceptibility of these isolates to 3 macrolide antibiotics was assessed using broth microdilution method. The *23S rRNA* genes of macrolide-resistant *M. pneumoniae* strains were sequenced, and the presence of target methylation genes (*ermA*, *ermB*, and *ermC*), efflux pump genes (*mefA*, *mefA/E*, *msrA*, and *msrA/B*), and the macrolide resistance gene *mphC* was identified through polymerase chain reaction (PCR) testing. Additionally, MICs were determined with and without the efflux pump inhibitor reserpine. A total of 72 *M. pneumoniae* strains were isolated from adult patients, with 41.7% (30/72) exhibiting macrolide resistance. Among the 3 macrolides tested, the 16-membered-ring midecamycin exhibited the greatest activity (MIC_90_: 16 µg/ml) against *M. pneumoniae*. All macrolide-resistant *M. pneumoniae* strains harbored mutations at the 2063 site in domain V of the *23S rRNA* gene. Two macrolide-resistant *M. pneumoniae* clinical isolates were found to harbor the efflux pump genes *msrA/B* and *mefA*. The efflux pump inhibitor reserpine reduced the MIC for azithromycin in these two strains to a quarter of their original values. In summary, macrolide-resistant *M. pneumoniae* is commonly observed among adults in Beijing. Point mutations are the primary mechanism responsible for macrolide resistance in adults with *M. pneumoniae*. Additionally, the efflux pump mechanism may contribute partially to this resistance. Midecamycin presents a promising alternative drug for treating *M. pneumoniae* infections, particularly in cases of azithromycin-resistant *M. pneumoniae* infection in young children.

## 1 Introduction

*Mycoplasma pneumoniae* is one of the most significant pathogens responsible for respiratory infections in humans. It is estimated to account for 10% to 30% of cases of community-acquired pneumonia (CAP) (Atkinson et al., 2008). Although *M. pneumoniae* infection is typically self-limiting, severe *M. pneumoniae* pneumonia has been increasingly reported in recent years (Cillóniz et al., 2016; Waites et al., 2017; Ha et al., 2023; Lai et al., 2024). *M. pneumoniae* is an atypical pathogen as it lacks a cell wall, rendering it innately resistant to a wide range of antimicrobial drugs that target the cell wall, such as β-lactams (Lee et al., 2018; Waites et al., 2017). Macrolides, fluoroquinolones, and tetracyclines are three major classes of antibiotics effective against *M. pneumoniae* (Waites et al., 2017; Gautier-Bouchardon, 2018). Macrolides are recommended as the first-line treatment for *M. pneumoniae* infection in adults and are preferred for children (Principi and Esposito, 2013; Qu et al., 2013). However, as the prescription of macrolide antibiotics for outpatients with CAP has increased, the acquired resistance of *M. pneumoniae* to macrolide antibiotics has gradually emerged in response to antibiotic selective pressure. Recent studies have demonstrated a significant worldwide increase in the prevalence of macrolide-resistant *M. pneumoniae*, with a particularly marked rise observed in Asia (Zhou et al., 2015; Waites et al., 2017). Recent reports from China indicate that the macrolide resistance rate of *M. pneumoniae* can be as high as 80% to 100% in various regions (Zhou et al., 2015; Zhao et al., 2019; Wang et al., 2022b, 2022a). Macrolide-resistant *M. pneumoniae* infections are more prevalent in children than in adults (Miyashita et al., 2012; Yan et al., 2020; Kim et al., 2022; Jiang et al., 2024), however, the disease burden caused by these infections in adults also warrants attention (Lai et al., 2024). How macrolides should be deployed in adult patients with *M. pneumoniae* infection, has become a matter of urgent concern in the clinical community.

Bacterial resistance to macrolides is mediated by various mechanisms, including modification of target sites by methylation or mutation in the 23S rRNA or large ribosomal subunit proteins, drug-inactivating and efflux of macrolides from bacterial cell resulting from efflux pump expression (Dinos, 2017). In *M. pneumoniae*, resistance to macrolides is primarily attributed to mutations in the domains V and/or II of *23S rRNA* (Gaynor and Mankin, 2003; Bébéar and Pereyre, 2005). Furthermore, mutations in ribosomal proteins L4 and L22 also play a role in conferring macrolide resistance in *M. pneumoniae* (Pereyre et al., 2004; Liu et al., 2014; Wang et al., 2023). Nevertheless, the resistance mechanisms of some resistant strains could not be fully explained by target mutations. *M. pneumoniae* and *Streptococcus pneumoniae* infections are prevalent causes of CAP in China, and co-infections with these two pathogens are also common (Cao et al., 2010). Target methylation modification represents the primary mechanism of macrolide resistance in *S. pneumoniae* in China (Zhao et al., 2020). In such cases, it remains unclear whether the target methylation genes are transferred from *S. pneumoniae* to *M. pneumoniae* through plasmids or other mobile genetic elements, conferring high levels of resistance to macrolides in *M. pneumoniae*. Furthermore, there has been no investigation into efflux pump mechanism in drug-resistant clinical isolates of *M. pneumoniae* in adults, and it remains unclear whether drug inactivation mechanisms are involved in macrolide resistance in *M. pneumoniae*.

We investigated the resistance of *M. pneumoniae* clinical isolates to macrolides in adult patients to guide the effective use of currently available macrolides. Furthermore, to elucidate the mechanisms of macrolide resistance in *M. pneumoniae* in adults, we performed an extensive analysis of target mutations, target methylation modifications, efflux pump activity, and drug-inactivating enzymes in resistant clinical isolates.

## 2 Materials and methods

### 2.1 Clinical *M. pneumoniae* isolates

All *M. pneumoniae* clinical strains were isolated from oropharyngeal samples of adult patients presenting with subacute cough and suspected *M. pneumoniae* infection with CAP. These samples were collected from both respiratory outpatient and inpatient departments of the Fifth Medical Center of PLA General Hospital from January 1, 2011, to June 30, 2017. Screening criteria for patients with subacute cough were similar to those described by Yuan et al., except for age ≥18 years (Yuan et al., 2014). The screening criteria for CAP patients with suspected *M. pneumoniae* infection were based on the rapid scoring system for *M. pneumoniae* Pneumonia of the Japanese Respiratory Society (JRS) with modifications (Ishida, et al., 2007). The presence of *M. pneumoniae* pneumonia is indicated by meeting four of the criteria in this scoring system, or three of the first five criteria. All strains were detected through culture and real-time quantitative polymerase chain reaction (PCR). *M. pneumoniae* was cultured using an established methodology (Waites et al., 2001). Positive cultures were identified by a color change from red to yellow in the broth medium (CM0403, OXOID, UK) and the presence of characteristic “fried egg” colonies on the agar medium (CM0401, OXOID, UK). A real-time quantitative PCR was used to quantify bacterial load by detecting the 16S rDNA of *M. pneumoniae*, utilizing primers and probe sequences as described previously (Yuan et al., 2014).

### 2.2 Antimicrobial susceptibility test

The *in vitro* susceptibility of the strains to 3 macrolide antibiotics (erythromycin, azithromycin, and midecamycin) was assessed using the broth microdilution method (Matsuoka et al., 2004). All 3 antibiotics were purchased from the National Institute for the Control of Pharmaceutical and Biological Products. A reference strain of *M. pneumoniae* designated FH (ATCC 15531), was used as the drug-sensitive control in this study. The minimum inhibitory concentration (MIC) for each agent was determined as the lowest concentration of each antimicrobial agent that prevented the color change, observed at the time when the growth controls first showed a color change (Waites et al., 2001). The MIC_50_ and MIC_90_ were defined as the MIC required to inhibit the growth of 50% and 90% of the subject bacteria, respectively, in a batch of tests. Each antimicrobial susceptibility test was performed in triplicate. Throughout the study, antimicrobial susceptibility tests were performed on all strains in strict accordance with the methodology described previously by Matsuoka et al (Matsuoka et al., 2004), and all referenced previous resistance breakpoint, the results were defined as resistant with a MIC of ≥32 µg/ml for erythromycin, azithromycin and midecamycin (Xin et al., 2009).

### 2.3 Polymerase chain reaction amplification and DNA sequencing

Total DNA was manually extracted using the QIAamp DNA Mini kit (QIAGEN, Germany). Primers were designed and synthesized based on GenBank and relevant literature (Matsuoka et al., 2003; Lu et al., 2010) to amplify *M. pneumoniae 5S rRNA*, *23S rRNA*, genes encoding target site-modifying rRNA methylases *ermA/B/C*, efflux pump genes *mefA*, *mefA/E*, *msrA*, and *msrA/B*, and the macrolide 2’-phosphotransferase *mphC*. The primer sequences are listed in **Supplementary Table 1**. The PCR products of *M. pneumoniae 5S rRNA*, *23S rRNA* genes were sequenced by INVITROGEN using a 3730XL DNA sequencer, and the PCR amplification products of *ermA/B/C*, *mefA*, *mefA/E*, *msrA*, *msrA/B*, and *mphC* genes were subjected to electrophoresis to visualize the target bands.

### 2.4 Effect of an efflux pump inhibitor (reserpine) on MICs

Reserpine was obtained from the National Institute for the Control of Pharmaceutical and Biological Products. MICs were assessed under 3 conditions: in the presence of each macrolide alone, the efflux pump inhibitor reserpine alone, and a combination of macrolides and reserpine. The microbroth dilution test methodology remains largely unchanged (Matsuoka et al., 2004), apart from a minor modification in the configuration of the 96-well plate. In summary, MICs were determined for each of the resistant strains in three distinct scenarios: exposure to macrolides alone, exposure to efflux pump inhibitor reserpine alone (20 µg/ml), and exposure to macrolides and reserpine (20 µg/ml).

## 3 Results

### 3.1 Antimicrobial susceptibility for *M. pneumoniae*

In this study, 72 strains of *M. pneumoniae* were isolated, and the MICs of all 72 clinical *M. pneumoniae* isolates, as well as standard control strain, were tested against 3 different macrolides. A total of 27 males and 45 females were included in the study, with an age range of 18-75 years. 26 specimens were derived from patients with CAP, while 46 were obtained from patients with subacute cough. The MICs of the standard strain FH (ATCC 15531) for the 3 tested agents were consistent with those of the standard strain M129 (ATCC 29342) provided by Dr. Waites KB, all were < 0.5µg/ml. Among the 3 macrolides, 41.7% (30/72) of the strains were resistant to erythromycin, the resistance rate to azithromycin was 38.9% (28/72), and 1.4% (1/72) strains showed resistance to midecamycin, with the following MIC values: Erythromycin (MIC_50_: < 0.5µg/ml, MIC_90_: ≥ 128 µg/ml), azithromycin (MIC_50_: <0.5µg/ml, MIC_90_: ≥ 128 µg/ml), midecamycin (MIC_50_: < 0.5µg/ml, MIC_90_: 16 µg/ml). 54 strains were obtained during the cold season, and 18 during the warm season. No significant difference was observed in resistance rates between strains from the two seasons (P = 0.408) (**Supplementary Table 2**). **Table 1** presents a detailed MIC distribution of the 3 agents tested against 30 resistance *M. pneumoniae*.

**Table 1:** Minimal inhibitory concentrations and the effect of efflux pump inhibitor reserpine on 30 resistant clinical isolates of *M. pneumonia*.

*Strain No.	MIC value (mg/L)					
	Erythromycin		Azithromycin		Midecamycin	
	alone	+ reserpine	alone	+ reserpine	alone	+ reserpine
S1 S2 S4 S8 S14 S17 S19 S21 C8 S23 C14 S30 S33 S34 C18 S36 C21 S38 C23 S39 S41 S43 S44 S45 S46 S53 C33 S61 S68 S73	128 32 64 64 128 128 64 128 128 64 64 64 128 128 64 64 128 128 128 128 128 128 128 128 128 128 128 128 128 128	128 32 64 64 128 128 64 128 128 64 64 64 128 128 64 64 128 128 128 128 128 128 128 128 128 128 128 128 128 128	128 <1 64 64 64 64 16 64 128 32 32 32 64 64 32 32 128 128 128 128 64 64 64 64 64 64 64 64 128 128	64 <1 32 32 32 32 16 32 32 32 16 16 16 32 16 16 64 64 64 64 64 32 32 32 32 32 32 32 128 64	16 1 8 8 8 8 2 8 16 8 8 8 8 8 8 8 16 16 16 16 8 8 8 8 8 8 8 8 16 32	16 1 8 8 8 8 2 8 8 8 8 8 8 8 8 8 16 16 16 16 8 8 8 8 8 8 8 8 16 32

*In the strain number, S indicates that the strains were from specimens of patients with subacute cough, while C indicates were from patients with CAP.

### 3.2 Target mutations and target modifications associated with macrolide resistance

**Figure 1:**
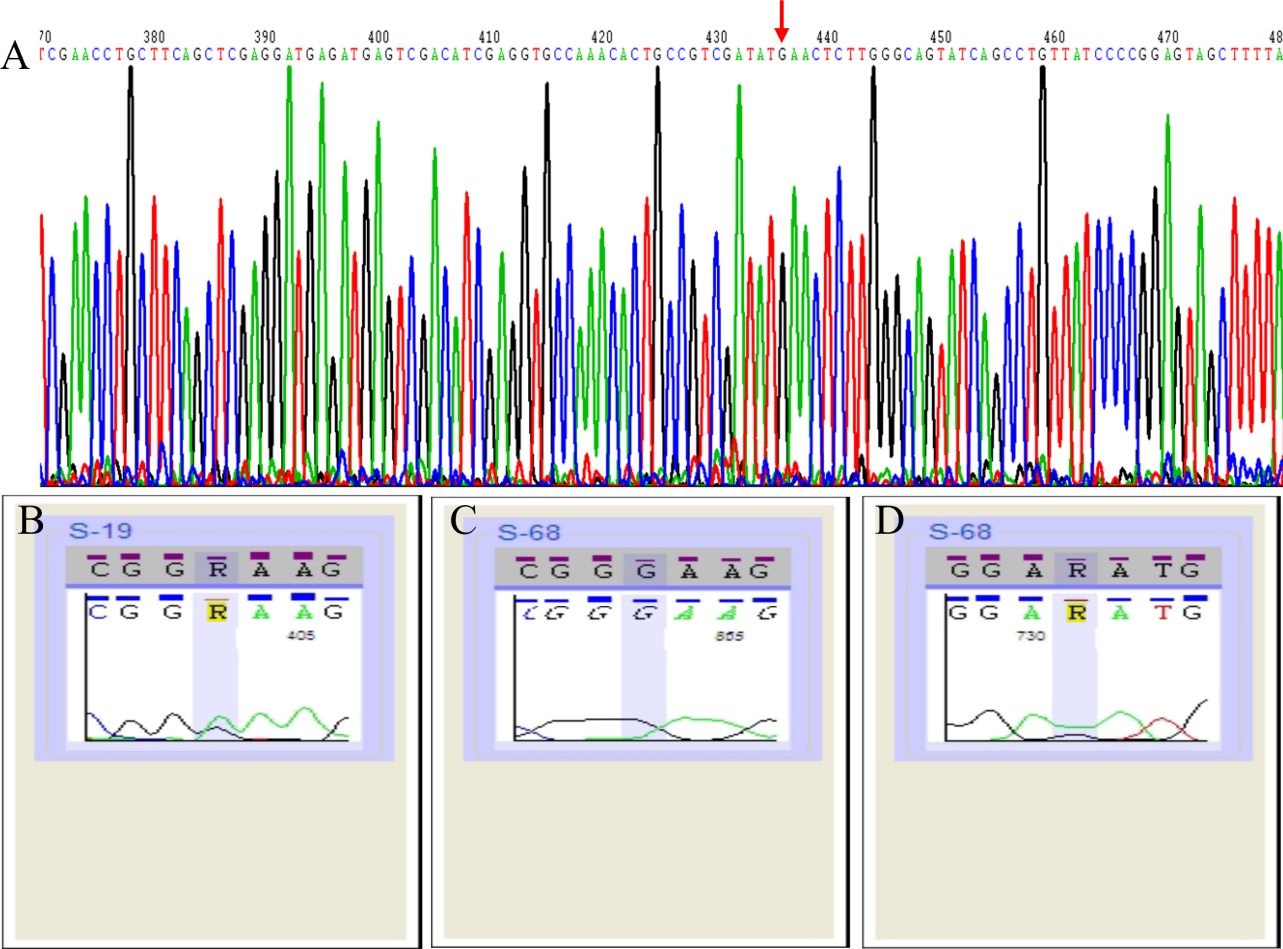
Schematic representation of 23S rRNA sequencing results of 30 macrolide-resistant *Mycoplasma pneumoniae* isolates. Red arrow indicate the A2063G point mutation in the gene sequence **(A)**. A2063R point mutation (heterozygote) in one strain (S19) **(B)**. A2063G mutation **(C)** and G648R mutation **(D)** in one strain (S68).

No mutations were detected in the *5S rRNA* of the 30 macrolide-resistant *M. pneumoniae* isolates. An A2063G point mutation was observed in the *23S rRNA* gene of 29 of these isolates (**Figure 1A**), and one strain (S19) exhibited an A-to-R transition at point 2063 (A2063R, heterozygote) (**Figure 1B**). In addition to an A2063G mutation, one strain (S68) harbored a G648R mutation (**Figures 1C, D**). Additionally, an A1029G mutation was identified in 30 macrolide-resistant clinical strains, the *M. pneumoniae* reference strain, and 30 macrolide-sensitive strains, indicating that this mutation is not associated with macrolide resistance. Moreover, the amplification of *ermA*, *ermB*, and *ermC* target methylation genes in 30 resistant *M. pneumoniae* strains failed to yield any gene products.

### 3.3 Efflux pump mechanism in *M. pneumoniae* isolates

**Figure 2:**
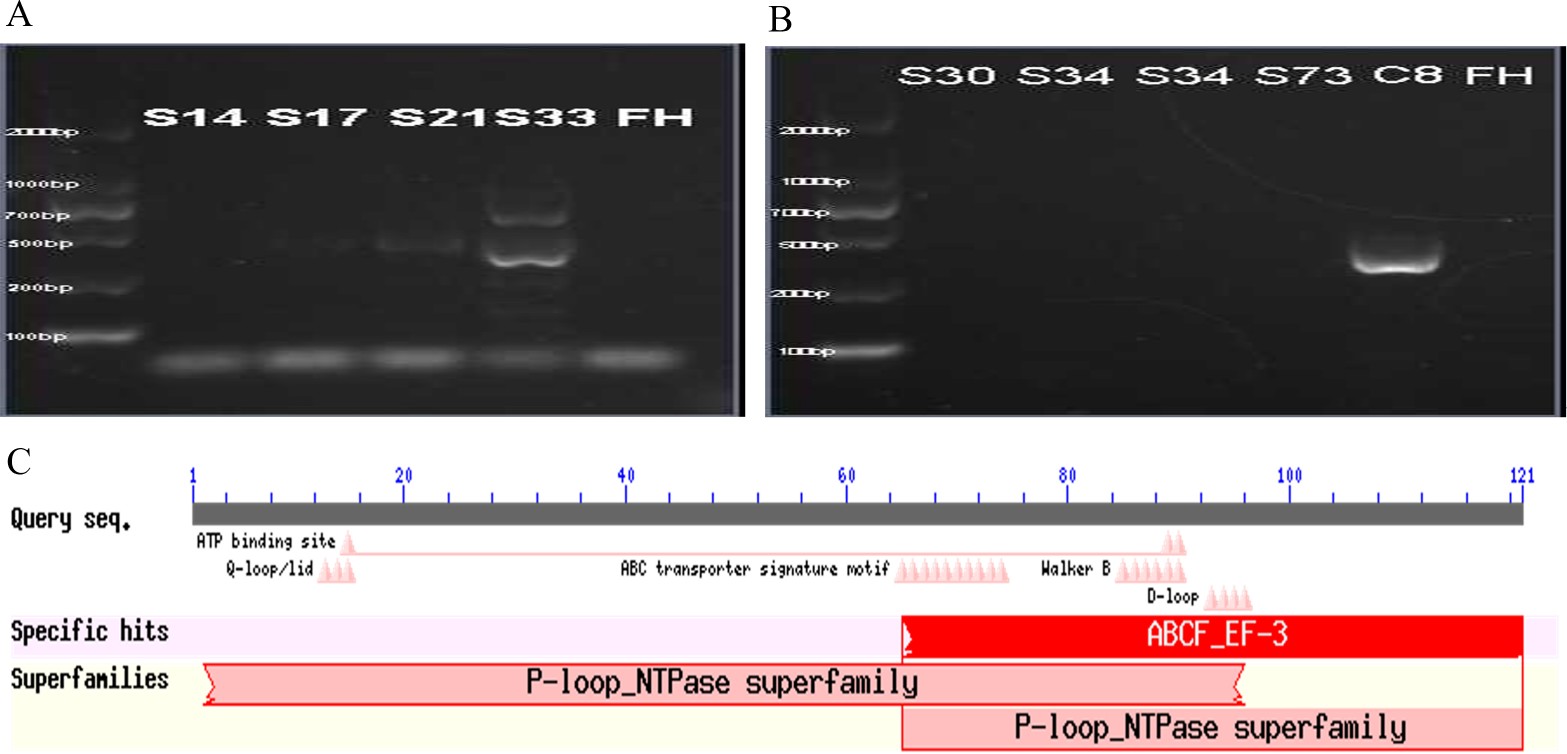
Detection of efflux pump genes. The PCR product (488 bp) of the *mefA* gene of one clinical macrolides-resistant *M. pneumoniae* strain **(A)**. The PCR product (399 bp) of *msrA/B* gene of one clinical macrolides-resistant *M. pneumoniae* strain **(B)**. The molecular weights labeled on the left side of the figure A and figure B are, from bottom to top, 100bp, 200bp, 600bp, 700bp, 1000bp and 2000bp. Results of conserved protein sequence comparison after efflux pump gene sequencing **(C)**.

To investigate the presence of an efflux pump, PCR amplification products for the corresponding efflux pump genes *mefA*, *mefA/E*, *msrA*, and *msrA/B* genes were subjected to electrophoresis to visualize the target bands. The target fragment for the *mefA* gene was 488 bp. Agarose gel electrophoresis revealed a 488 bp fragment, indicating successful amplification from one sample of macrolide-resistant *M. pneumoniae* clinical strains (**Figure 2A**). For the *msrA/B* gene (target fragment size 399 bp), one sample was found to amplify a 399 bp fragment (**Figure 2B**). BLAST analysis of the PCR product revealed a 99% similarity to the *msrC* gene (405 bp), which encodes an ABC (ATP-binding cassette) transporter protein in *Enterococcus faecium* (GeneBank: AJ243209.1), and a 95% similarity to the macrolide resistance-like protein gene (GeneBank: AY004350.1; 2479 bp) acquired from *E. faecium* strain TX2465 (**Supplementary Table 3**). Additionally, the protein sequence corresponding to this gene was found to be homologous to the P-loop_NTPase superfamily (**Figure 2C**), showing 99% similarity to the ABC transporter protein (ZP_00603470.1) and the acquired macrolide-resistant protein (AAF91071.1) in *E. faecium*.

In order to evaluate the potential role of the efflux pump in macrolide resistance, MICs were determined with and without the efflux pump inhibitor reserpine. Reserpine alone did not inhibit the growth of *M. pneumoniae* and did not affect the MICs for the three macrolides in the reference strain FH. Although reserpine did not change the MIC for erythromycin in the 30 macrolide-resistant clinical isolates, it reduced the MIC for azithromycin in 2 strains which harbored efflux pump genes to a quarter, in 23 of these strains to half of their original values, and the remaining 3 strains unchanged. Additionally, in one macrolide-resistant clinical isolate, reserpine decreased the MIC for midecamycin to half of its original value (Tabe 1).

### 3.4 Macrolide passivating enzymes in *M. pneumoniae* isolates

The PCR products of the resistant *M. pneumoniae* strains were subjected to agarose gel electrophoresis, which revealed the absence of macrolide 2’-phosphotransferase *mphC*.

## 4 Discussion

In the present study, we measured the resistance of *M. pneumoniae* to macrolides and observed a relatively high prevalence of macrolide resistance to *M. pneumoniae* in adult patients with *M. pneumoniae* infections in Beijing. An A to G mutation at the 2063 site in domain V of the *23S rRNA* gene was identified in all resistant isolates. Interestingly, we also identified the presence of the efflux pump gene. Furthermore, midecamycin demonstrated higher susceptibility to *M. pneumoniae* among the three macrolides.

The macrolide resistance rate in *M. pneumoniae* in adults was found to be 41.7% in our study, which is lower than the rates reported in most other studies conducted in China. Over the past two decades, investigations into macrolide-resistant *M. pneumoniae* in children have demonstrated the highest rates of resistance in some East Asian regions, with resistance rates reaching 81.6% (493/604 specimens) in Japan and up to 100% (49/49 specimens) in some parts of China (Tanaka et al., 2017; Zhao et al., 2019; Wang et al., 2022b, 2022a). Although macrolide resistance in *M. pneumoniae* is relatively low in Europe and the United States, with reported rates of 10% (10/114 specimens) in the United States (Rothstein et al., 2022) and 1% to 25% in Europe (Álvaro Varela et al., 2023) in children, resistance rates are on the rise in these regions as well. The disparate rates of macrolide resistance observed across different countries may be attributed to variations in the frequency of macrolide utilization, and there is a well-quantified correlation between antibiotic usage and the emergence of resistance (Klein et al., 2018; Bell et al., 2014). These findings indicate the necessity for heightened surveillance of macrolide-resistant *M. pneumoniae*, particularly in China. However, there is a paucity of data regarding the prevalence of macrolide-resistant *M. pneumoniae* in adults. Cao et al. reported a 69% macrolide resistance rate in *M. pneumoniae* among adults in China in 2010 (Cao et al., 2010); Yin et al. showed an 80% resistance rate to erythromycin in adult CAP isolates from three different Chinese cities between 2010 and 2012 (Yin et al., 2017). Zhou et al. observed a 100% macrolide resistance in *M. pneumoniae* isolates from adult patients with CAP in Zhejiang Province of China from 2012 to 2014 (Zhou et al., 2015); Jiang et al. investigated 41 *M. pneumoniae*-positive samples in Beijing, China, and found that only 10.5% exhibited the A2063G resistance mutation (Jiang et al., 2024). Our results contribute further data on macrolide resistance in *M. pneumoniae* among adults in China, demonstrating a lower rate of macrolide resistance than that reported in the majority of domestic studies. This discrepancy may be attributed to the varied sources of strains. *M. pneumoniae* has been shown to be highly prevalent among patients with subacute cough, as indicated by our previous study (Yuan et al., 2014). In our study, most isolates were obtained from patients presenting with subacute cough. Some of these patients had only been administered oral β-lactam antibiotics during the course of their illness, which had minimal impact on *M. pneumoniae*. Consequently, the probability of developing induced resistance was diminished.

The resistance of *M. pneumoniae* to macrolides does not appear to be reflected in resistance to all macrolide antibiotics. Our data revealed that erythromycin and azithromycin exhibited reduced activity against *M. pneumoniae*, whereas midecamycin showed good activity. Midecamycin, a 16-membered-ring macrolide, has shown *in vitro* activity against erythromycin-resistant *Streptococcus pyogenes* (Schlegel et al., 2001). In *in vitro* studies, the MIC of acetylmidecamycin (diacetate of midecamycin) was found to be considerably lower than that of other macrolide antibiotics, indicating a more potent activity for *M. pneumoniae* activity of acetylmidecamycin (Pereyre et al., 2001; Wang et al., 2020). Furthermore, the dosage and safety of midecamycin have been established in pediatric patients in a variety of countries (Wang et al., 2023; Kikuchi et al., 1979; Yoshida et al., 1982; Morikawa et al., 1994). *In vitro* resistance induction experiments have shown that screening for *M. pneumoniae* mutant strains of midecamycin is more difficult compared to other macrolide antibiotics (Wang et al., 2023). Additionally, strains resistant to midecamycin remained susceptible to 14- and 15-membered-ring macrolide antibiotics (Wang et al., 2023). Macrolides remain the most commonly utilized antibiotics for the treatment of *M. pneumoniae* infections in clinical practice. The frequency of antibiotic use is associated with the emergence of drug resistance. In China, erythromycin and azithromycin are more widely used than midecamycin, which may account for the higher MIC values observed for the former antibiotics. Since the MIC of midecamycin against *M. pneumoniae* is lower than that of other 14- or 15-membered-ring macrolides, midecamycin may serve as a promising treatment option for *M. pneumoniae* infections, particularly in children.

Macrolide antibiotics bind to specific nucleotides in structural domains II and/or V of *23S rRNA* in the 50S bacterial ribosomal subunit, thereby blocking protein synthesis by causing premature dissociation of the peptidyl-tRNA from the ribosome and achieving antimicrobial efficacy. Specific mutations in binding sites result in a reduction in the binding of drugs to *M. pneumoniae*, ultimately conferring resistance to macrolides (Vázquez-Laslop and Mankin, 2018; Dinos, 2017). In *M. pneumoniae*, the A2063G mutation, which is the primary mutation responsible for resistance to macrolides, is situated within the peptidyl-transferase center of the 23S rRNA V region (Gaynor and Mankin, 2003; Bébéar and Pereyre, 2005). This structural domain has been demonstrated to serve as a binding site for macrolide antibiotics (Jelić and Antolović, 2016). The A2063 locus mutations were identified in all *M. pneumoniae* strains exhibiting macrolide resistance in our experiment. These findings are consistent with previous studies that point mutations in the peptidyl transferase loop of the *23S rRNA* in *M. pneumoniae*, such as A2063G/T/C, C2617G, A2064G/C, and A2067G, are key contributors to macrolide resistance (Lucier et al., 1995; Bébéar and Pereyre, 2005; Suzuki et al., 2013; Waites et al., 2017). The 2063 site point mutation is capable of causing high levels of resistance to 14- and 15-membered-ring macrolides in *M. pneumoniae*. For the 16-membered-ring macrolides, mutations at sites 2063 and 2064 were found to be associated with low to moderate levels of resistance. Whereas, the A2067G mutation resulted in the highest level of resistance due to its ability to form a specific covalent bond with the 16-membered ring (Cardinale et al., 2011; Principi and Esposito, 2013). The differences in sensitivity may be attributed to variations in the binding sites, drug orientation, and binding kinetics between 16-membered-ring macrolide antibiotics and 14- and 15-membered-ring macrolide antibiotics (Starosta et al., 2010; Wang et al., 2023). However, no mutations were observed at locus 2067 in our study. Antimicrobial susceptibility testing results of clinical isolates for midecamycin also demonstrated that mutations at locus A2063 had a minimal effect on susceptibility to the 16-membered-ring macrolides. Most A2063G mutant strains were either susceptible to midecamycin or showed only a low level of resistance. This supports the potential of midecamycin as a promising alternative agent for treating *M. pneumoniae* infections. However, one strain was highly resistant to midecamycin (MIC >32 mg/L), indicating that mechanisms beyond target mutation may contribute to resistance against 16-membered-ring macrolides.

Active efflux mechanism plays a role in macrolide resistance (Ma et al., 2024). Of particular interest, we further screened for common efflux pump genes and identified the presence of *mefA* and *msrA/B* genes. Sequencing of the *msrA/B* gene in this resistant strain revealed a high degree of similarity between the gene and the *msrC* gene, which encodes an ABC transporter protein in *E. faecium*. The protein encoded by this *msrA/B* gene is also similar to the *E. faecium*-acquired macrolide-resistant protein, which is absent in the reference strain. Efflux pump inhibitor reserpine was used to assess the role of efflux pumps in macrolide resistance in *M. pneumoniae*. Our findings revealed that for 2 resistance strains in which the efflux pump gene was identified, the MICs of azithromycin and midecamycin were reduced to half to a quarter of their original values; for 23 resistance strains, the MICs were reduced to half; and for other 3 resistance strains, no change was observed. The disparate effects on the MICs may be attributed to the following factors: Firstly, a not significant multiplicative change in MIC with the addition of an efflux pump inhibitor may not be indicative of the presence of an efflux pump in a strain (Baron and Rolain, 2018); secondly, the expression levels of efflux pump genes in these strains were different (Heijden et al., 2023); thirdly, there may be the potential existence of additional resistance mechanisms beyond the *23S rRNA* point mutation and the efflux pump effect. Further studies are necessary to substantiate these hypotheses. Six bacterial drug efflux pump families have been identified as being involved in the efflux pathway (Du et al., 2018; Zhang et al., 2024). The efflux pump *msrA/B* gene identified in our study may belong to the ATP-binding cassette (ABC) family. Reserpine is known to inhibit multiple drug resistance (MDR) efflux pumps, including ATP-dependent efflux pumps (Frempong-Manso et al., 2009; Huang et al., 2013; Li et al., 2017). Additionally, the small multidrug resistance (SMR) family has been linked to macrolide efflux. However, it remains uncertain whether the 23 strains exhibiting halved MICs possess other efflux pumps like SMR family. These findings suggest the involvement of an efflux pump system mechanism, possibly an ABC transporter, may play an important role in promoting macrolide resistance, thereby providing further insight into the mechanisms of macrolide resistance in *M. pneumoniae*.

There are several limitations to our study. Firstly, a relatively small sample size affects the generalizability of the findings. Variations in subject populations may have contributed to discrepancies in observed resistance rates. Secondly, our study did not include subgroup analysis to compare differences in resistance between clinical isolates from patients with subacute cough and those with pneumonia. Thirdly, this study did not undertake a more in-depth exploration of resistance mechanisms through the use of whole genome sequencing and bioinformatics tools. Additionally, the resistance criteria were based on the 2006 CLSI standards, which may have led to discrepancies when compared to those of more recent studies. As the present study was initiated prior to the release of the most recent guideline on methods for antimicrobial susceptibility testing for *M. pneumoniae* and was in strict accordance with the already developed method throughout the study, reference was made to previous resistance breakpoints. Despite these limitations, our study highlights the prevalence of macrolide-resistant *M. pneumoniae* in adults and supports the hypothesis that efflux pump genes contribute to macrolide resistance in clinical *M. pneumoniae* isolates.

In conclusion, this study presented valuable insights on *M. pneumoniae* resistance to macrolides in adults. Macrolide-resistant *M. pneumoniae* is highly prevalent in adults. Resistance is primarily attributed to mutations in domain V of the *23S rRNA* gene, and presence of efflux pump may also bring about the resistance phenotype. The observed reduction in MICs for azithromycin against macrolides-resistant *M. pneumoniae* in the presence of reserpine suggests that reserpine might be a promising candidate for combination therapy. Additionally, our findings support the potential of midecamycin as an effective alternative for the treatment of macrolide-resistant *M. pneumoniae* infections.

## Data Availability

The raw data supporting the conclusions of this article will be made available by the authors, without undue reservation.
