# Supplementary material for: Macrolide resistance in Mycoplasma pneumoniae in adult patients
Source: Front Cell Infect Microbiol. 2025 Mar 4;15:1496521. doi: 10.3389/fcimb.2025.1496521 (PMC11955812; doi:10.3389/fcimb.2025.1496521)
Supplement: Supplementary file 1 [file Table1.docx]

**Supplementary Table 1. Sequences of primers used in this study**

| **Primer name** | **Primer sequence (5’-3’)** | **Product length** |
| --- | --- | --- |
| 5S rRNA | Forward: GGAAATACGCAGGATTGTCG  Reverse: CAAGTCCCATTCTGCCCCTA | 378bp |
| 23S rRNA 1 | Forward: AGCGGATGACTTGTGATTAGGG  Reverse: CGTTCTTCACTGCGGCTTACTT | 1137bp |
| 23S rRNA 2 | Forward: AGCGGATGACTTGTGATTAGGG  Reverse: CGTTCTTCACTGCGGCTTACTT | 982bp |
| 23S rRNA 3 | Forward: AACCAGTACCGAGAACGAACAC  Reverse: GACATGGGCACCAAACAAAA | 1330bp |
| *erm*A | Forward: TCTAAAAAGCATGTAAAAGAA  Reverse: CTTCGATAGTTTATTAATATTAGT | 645bp |
| *erm*B | Forward: GAAAAAGTACTCAACCAAATA  Reverse: AGTAATGGTACTTAAATTGTTTAC | 639bp |
| *erm*C | Forward: TCAAAACATAATATAGATAAA  Reverse: GCTAATATTGTTTAAATCGTCAAT | 642bp |
| *mef*A/E | Forward: AGTATCATTAATCACTAGTGC  Reverse: TTCTTCTGGTACTAAAAGTGG | 348bp |
| *msr*A/B | Forward: GCAAATGGTGTAGGTAAGACAACT  Reverse: ATCATGTGATGTAAACAAAAT | 399bp |
| *MefA* | Forward: AAGCAGAACCCAAATAACG  Reverse: AGCCAAGATAGGTAACCATAGA | 488bp |
| *msr*A | Forward: ACAATTTATCTTGCTGGTGG  Reverse: ACTGCTTCAGCGTGTAGAG | 2758bp |
| *mph*C | Forward: ACAACCGACAGTATGAGTGG  Reverse: ATCTTTTTTGTTCATTATAA | 2963bp |

**Supplementary Table 2. Clinical information of the patients**

|  | **Patients (N=72)** |
| --- | --- |
| **Age (years)**  **Sex**  Male  Female  **Sample source**  Subacute cough  CAP  **Season**  Cold  Warm | 39.5±13.6  27 (37.5%)  45 (62.5%)  46 (63.9%)  26 (36.1%)  *(P=0.408)  54 (75.0%)  18 (25.0%) |

Age is described as mean ± standard deviation, the rest are described as n (%). The sampling date was divided into cold season or warm season based on the climate characteristics, that is, the 6 months with the highest monthly average temperature was the warm season, and other months were the cold season. *(P=0.408) indicates that resistance rates were compared between strains collected in the cold and warm seasons.

**Supplementary Table 3. Results of *msr*A/B gene sequencing comparison**

| **Accession** | **Description** | [**Max score**](http://blast.ncbi.nlm.nih.gov/Blast.cgi?CMD=Get&ALIGNMENTS=100&ALIGNMENT_VIEW=Pairwise&DATABASE_SORT=0&DESCRIPTIONS=100&FIRST_QUERY_NUM=0&FORMAT_OBJECT=Alignment&FORMAT_PAGE_TARGET=&FORMAT_TYPE=HTML&GET_SEQUENCE=yes&I_THRESH=&MASK_CHAR=2&MASK_COLOR=1&NEW_VIEW=yes&NUM_OVERVIEW=100&OLD_BLAST=false&PAGE=MegaBlast&QUERY_INDEX=0&QUERY_NUMBER=0&RESULTS_PAGE_TARGET=&RID=UGZNK51W01S&SHOW_LINKOUT=yes&SHOW_OVERVIEW=yes&STEP_NUMBER=&DISPLAY_SORT=1&HSP_SORT=1#sort_mark) | [**Total score**](http://blast.ncbi.nlm.nih.gov/Blast.cgi?CMD=Get&ALIGNMENTS=100&ALIGNMENT_VIEW=Pairwise&DATABASE_SORT=0&DESCRIPTIONS=100&FIRST_QUERY_NUM=0&FORMAT_OBJECT=Alignment&FORMAT_PAGE_TARGET=&FORMAT_TYPE=HTML&GET_SEQUENCE=yes&I_THRESH=&MASK_CHAR=2&MASK_COLOR=1&NEW_VIEW=yes&NUM_OVERVIEW=100&OLD_BLAST=false&PAGE=MegaBlast&QUERY_INDEX=0&QUERY_NUMBER=0&RESULTS_PAGE_TARGET=&RID=UGZNK51W01S&SHOW_LINKOUT=yes&SHOW_OVERVIEW=yes&STEP_NUMBER=&DISPLAY_SORT=2&HSP_SORT=1#sort_mark) | [**Query coverage**](http://blast.ncbi.nlm.nih.gov/Blast.cgi?CMD=Get&ALIGNMENTS=100&ALIGNMENT_VIEW=Pairwise&DATABASE_SORT=0&DESCRIPTIONS=100&FIRST_QUERY_NUM=0&FORMAT_OBJECT=Alignment&FORMAT_PAGE_TARGET=&FORMAT_TYPE=HTML&GET_SEQUENCE=yes&I_THRESH=&MASK_CHAR=2&MASK_COLOR=1&NEW_VIEW=yes&NUM_OVERVIEW=100&OLD_BLAST=false&PAGE=MegaBlast&QUERY_INDEX=0&QUERY_NUMBER=0&RESULTS_PAGE_TARGET=&RID=UGZNK51W01S&SHOW_LINKOUT=yes&SHOW_OVERVIEW=yes&STEP_NUMBER=&DISPLAY_SORT=4&HSP_SORT=0#sort_mark) | [**E value**](http://blast.ncbi.nlm.nih.gov/Blast.cgi?CMD=Get&ALIGNMENTS=100&ALIGNMENT_VIEW=Pairwise&DATABASE_SORT=0&DESCRIPTIONS=100&FIRST_QUERY_NUM=0&FORMAT_OBJECT=Alignment&FORMAT_PAGE_TARGET=&FORMAT_TYPE=HTML&GET_SEQUENCE=yes&I_THRESH=&MASK_CHAR=2&MASK_COLOR=1&NEW_VIEW=yes&NUM_OVERVIEW=100&OLD_BLAST=false&PAGE=MegaBlast&QUERY_INDEX=0&QUERY_NUMBER=0&RESULTS_PAGE_TARGET=&RID=UGZNK51W01S&SHOW_LINKOUT=yes&SHOW_OVERVIEW=yes&STEP_NUMBER=&DISPLAY_SORT=0&HSP_SORT=0#sort_mark) | [**Max ident**](http://blast.ncbi.nlm.nih.gov/Blast.cgi?CMD=Get&ALIGNMENTS=100&ALIGNMENT_VIEW=Pairwise&DATABASE_SORT=0&DESCRIPTIONS=100&FIRST_QUERY_NUM=0&FORMAT_OBJECT=Alignment&FORMAT_PAGE_TARGET=&FORMAT_TYPE=HTML&GET_SEQUENCE=yes&I_THRESH=&MASK_CHAR=2&MASK_COLOR=1&NEW_VIEW=yes&NUM_OVERVIEW=100&OLD_BLAST=false&PAGE=MegaBlast&QUERY_INDEX=0&QUERY_NUMBER=0&RESULTS_PAGE_TARGET=&RID=UGZNK51W01S&SHOW_LINKOUT=yes&SHOW_OVERVIEW=yes&STEP_NUMBER=&DISPLAY_SORT=3&HSP_SORT=3#sort_mark) |
| --- | --- | --- | --- | --- | --- | --- |
| [AJ243209.1](http://www.ncbi.nlm.nih.gov/nucleotide/6687492?report=genbank&log$=nucltop&blast_rank=1&RID=UGZNK51W01S) | Enterococcus faecium partial *msr*C gene for ABC transporter protein | [732](http://blast.ncbi.nlm.nih.gov/Blast.cgi#6687492#6687492) | 732 | 98% | 0.0 | 99% |
| [CP003351.1](http://www.ncbi.nlm.nih.gov/nucleotide/378937014?report=genbank&log$=nucltop&blast_rank=2&RID=UGZNK51W01S) | Enterococcus faecium Aus0004, complete genome | [723](http://blast.ncbi.nlm.nih.gov/Blast.cgi#378937014#378937014) | 723 | 97% | 0.0 | 99% |
| [DQ384855.1](http://www.ncbi.nlm.nih.gov/nucleotide/88770353?report=genbank&log$=nucltop&blast_rank=3&RID=UGZNK51W01S) | Enterococcus faecium strain FAIR-E 3 MsrC (*msr*C) gene, partial cds | [697](http://blast.ncbi.nlm.nih.gov/Blast.cgi#88770353#88770353) | 697 | 93% | 0.0 | 99% |
| [AY004350.1](http://www.ncbi.nlm.nih.gov/nucleotide/10442769?report=genbank&log$=nucltop&blast_rank=4&RID=UGZNK51W01S) | Enterococcus faecium strain TX2465 acquired macrolide resistance-like protein gene, complete cds | [640](http://blast.ncbi.nlm.nih.gov/Blast.cgi#10442769#10442769) | 640 | 97% | 3e-180 | 95% |
| [DQ384856.1](http://www.ncbi.nlm.nih.gov/nucleotide/88770355?report=genbank&log$=nucltop&blast_rank=5&RID=UGZNK51W01S) | Enterococcus faecium strain FAIR-E 349 MsrC (*msr*C) gene, partial cds | [627](http://blast.ncbi.nlm.nih.gov/Blast.cgi#88770355#88770355) | 627 | 93% | 3e-176 | 96% |
| [AF313494.1](http://www.ncbi.nlm.nih.gov/nucleotide/12659043?report=genbank&log$=nucltop&blast_rank=6&RID=UGZNK51W01S) | Enterococcus faecium strain TX1330 MsrC gene, complete cds | [617](http://blast.ncbi.nlm.nih.gov/Blast.cgi#12659043#12659043) | 617 | 95% | 2e-173 | 95% |
| [HQ651921.1](http://www.ncbi.nlm.nih.gov/nucleotide/340509495?report=genbank&log$=nucltop&blast_rank=7&RID=UGZNK51W01S) | Enterococcus durans strain Kov-3E MsrC (*msr*C) gene, partial cds | [555](http://blast.ncbi.nlm.nih.gov/Blast.cgi#340509495#340509495) | 555 | 81% | 1e-154 | 96% |
| [HQ651922.1](http://www.ncbi.nlm.nih.gov/nucleotide/340509497?report=genbank&log$=nucltop&blast_rank=8&RID=UGZNK51W01S) | Lactobacillus fermentum strain RM-3-2 MsrC (*msr*C) gene, partial cds | [538](http://blast.ncbi.nlm.nih.gov/Blast.cgi#340509497#340509497) | 538 | 81% | 1e-149 | 96% |
| [HQ651920.1](http://www.ncbi.nlm.nih.gov/nucleotide/340509493?report=genbank&log$=nucltop&blast_rank=9&RID=UGZNK51W01S) | Enterococcus casseliflavus strain ABM-1 MsrC (*msr*C) gene, partial cds | [538](http://blast.ncbi.nlm.nih.gov/Blast.cgi#340509493#340509493) | 538 | 81% | 1e-149 | 96% |
| [HQ651919.1](http://www.ncbi.nlm.nih.gov/nucleotide/340509491?report=genbank&log$=nucltop&blast_rank=10&RID=UGZNK51W01S) | Pediococcus pentosaceus strain CHS-3E MsrC (*msr*C) gene, partial cds | [527](http://blast.ncbi.nlm.nih.gov/Blast.cgi#340509491#340509491) | 527 | 81% | 3e-146 | 95% |
| [DQ681199.1](http://www.ncbi.nlm.nih.gov/nucleotide/110816303?report=genbank&log$=nucltop&blast_rank=11&RID=UGZNK51W01S) | Streptococcus thermophilus strain H erythromycin resistance (*msr*C) gene, partial cds | [442](http://blast.ncbi.nlm.nih.gov/Blast.cgi#110816303#110816303) | 442 | 63% | 1e-120 | 97% |
| [DQ681198.1](http://www.ncbi.nlm.nih.gov/nucleotide/110816301?report=genbank&log$=nucltop&blast_rank=12&RID=UGZNK51W01S) | Streptococcus thermophilus strain NCDC 217 erythromycin resistance (*msr*C) gene, partial cds | [398](http://blast.ncbi.nlm.nih.gov/Blast.cgi#110816301#110816301) | 398 | 57% | 2e-107 | 97% |
